# ﻿A new species of Simulium (Gomphostilbia) (Diptera, Simuliidae) from Thailand, with a key to identify females of 14 species of the *Simuliumvaricorne* species-group

**DOI:** 10.3897/zookeys.1083.77428

**Published:** 2022-01-21

**Authors:** Wichai Srisuka, Kittipat Aupalee, Yasushi Otsuka, Masako Fukuda, Hiroyuki Takaoka, Atiporn Saeung

**Affiliations:** 1 Entomology Section, Queen Sirikit Botanic Garden, P.O. Box 7, Maerim, Chiang Mai 50180, Thailand Queen Sirikit Botanic Garden Chiang Mai Thailand; 2 Center of Insect Vector Study, Department of Parasitology, Faculty of Medicine, Chiang Mai University, Chiang Mai 50200, Thailand Chiang Mai University Chiang Mai Thailand; 3 Research Center for the Pacific Islands, Kagoshima University, Korimoto 1–21–24, Kagoshima City, Kagoshima 890–8580, Japan Kagoshima University Kagoshima Japan; 4 Institute for Research Management, Oita University, Idaigaoka 1–1, Hasama, Yufu City, Oita, 879–5593, Japan Oita University Yufu Japan; 5 Higher Institution of Centre of Excellence (HICoE), Tropical Infectious Diseases Research and Education Centre, (TIDREC), Universiti Malaya, Kuala Lumpur, 50603, Malaysia Universiti Malaya Kuala Lumpur Malaysia

**Keywords:** Aquatic insects, biodiversity, blackflies, sweeping net, taxonomy

## Abstract

Simulium (Gomphostilbia) khelangense**sp. nov.** is described on the basis of females, collected by a sweeping net in Lampang, Phitsanulok and Chiang Mai Provinces, Thailand. This new species is placed in the *S.chumpornense* subgroup of the *S.varicorne* species-group in the subgenus Gomphostilbia Enderlein by having the antenna with eight flagellomeres, pleural membrane bare, and female subcosta lacking hairs. It is similar to *S.kuvangkadilokae* Pramual & Tangkawanit from Thailand in the same subgroup but is barely distinguished from the latter species by the head width relative to the greatest width of the frons and length of the labrum relative to the clypeus. A genetic analysis using the COI gene sequences similarly shows that *S.khelangense***sp. nov.** is most closely related to *S.kuvangkadilokae*, with a genetic distance of 1.23–2.81%. A revised key to identify females of 14 species of the *S.varicorne* species-group is provided.

## ﻿Introduction

The *Simuliumvaricorne* species-group, one of the 15 species-groups of the subgenus Gomphostilbia Enderlein of the genus *Simulium* Latreille, redefined by Takaoka (2012), is small, consisting of 14 species, of which 12 have been recorded in the Oriental region and the remaining two in the Palearctic region (Adler 2021). In Thailand, five species of this group are recorded: *Simuliumburtoni* Takaoka & Davies, *S.chumpornense* Takaoka & Kuvangkadilok, *S.kuvangkadilokae* Pramual & Tangkawanit, *S.novemarticulatum* Takaoka & Davies, and *S.piroonae* Takaoka & Srisuka (Kuvangkadilok and Takaoka 2000; Takaoka and Choochote 2004; Pramual and Tangkawanit 2008; Takaoka et al. 2010, 2014).

Biting habits and other biological aspects of these species remain unknown, although females of *S.burtoni* and *S.chumpornense* were captured using human attractants at low and medium elevations in Doi Inthanon National Park in Chiang Mai (Choochote et al. 2005), and females of *S.chumpornense* were natural vectors of protozoan parasites of the genera *Leucocytozoon* Berestneff and *Trypanosoma* Gruby (Thaijarern et al. 2019; Pramual et al. 2020).

Recently, we found a female of an unnamed species of the *S.varicorne* species-group, for which hereafter we call “*Simulium* sp.” as used by Aupalee et al. (2020), when morphologically and molecularly investigating parasites in adult female black flies collected by a sweeping net at Ban Pang Dang, Chiang Mai Province, Thailand. An unknown filarial species (probably a new species) was found in this unnamed species (Aupalee et al. 2020). *Simulium* sp. is placed in the *S.chumpornense* subgroup in the same species-group by lacking hairs ventrally on the subcosta, as defined by Takaoka (2012). It is morphologically similar to *S.kuvangkadilokae* of the same subgroup by having the hind tibia darkened on the apical half (Takaoka et al. 2014) and also genetically close to the latter species with a genetic distance of 1.99–2.36% (Aupalee et al. 2020).

In this study, we aimed to evaluate the status of *S.* sp. by morphologically and molecularly examining additional adult females collected by a sweeping net while they were flying around a human attractant, and to provide a revised key to identify females of 14 species of the *S.varicorne* species-group.

## ﻿Materials and methods

### ﻿Morphological analysis

Nine females of adult black flies (with eight antennal flagellomeres and without hairs ventrally on their subcosta) preserved in 80% ethanol after collection at three localities were used in this study. All were morphologically examined for color of legs, and heads and abdomens of three females (from each site) were treated with KOH solution overnight and observed in detail. The methods of collection, description and illustration, as well as terms for morphological features, followed those of Takaoka (2003). The type specimens are deposited at the Entomology Section, Queen Sirikit Botanic Garden, Chiang Mai, Thailand.

All but two were separated into three parts, head, thorax, and abdomen, and the thoraces were used for DNA analysis. The localities, number of females, designated numbers for DNA analysis are as follows:

- Site 1 at Pratoo Pha, Mueang, Lampang Province: three females (CPPT–1, CPPT–2, CPPT–3)
- Site 2 at Ban Lek, Fang District, Chiang Mai Province: three females (CPPH-1, two females not dissected)
- Site 3 at Ban Romklao Botanic Garden, Chat Trakan, Phitsanulok Province: three females (CPRK–1, CPRK–2, CPRK–3).

### ﻿Genetic analysis

The procedures for DNA extraction, PCR amplification, and sequencing followed those of Aupalee et al. (2020). In brief, total DNA was extracted from the thorax of individual adult black flies, using the Gene JET Genomic DNA Purification Kit (Thermo Fisher Scientific, Waltham, MA). DNA amplification of the COI gene using the LCO1490 forward primer and HCO2198 reverse primer (Folmer et al. 1994) was carried out with a reaction mixture of 20 μl consisting of 2 μl of DNA template, 0.5 U of *Taq* DNA polymerase, 3 mM of MgCl_2_, 0.25 mM dNTPs and 0.2 μM of each primer. The thermal cycling for PCR was as follow: 94 °C for 2 min followed by 40 cycles of 94 °C for 30 sec, 50 °C for 45 sec, and 72 °C for 45 sec, with a final extension at 72 °C for 5 min. After PCR amplification, the amplified products were subjected to electrophoresis on 1.5% agarose gel, stained with Ultrapower (BioTeke, Beijing, China) dye, and 100 bps DNA marker was used as standard. PCR products were purified and sequenced using the BigDyeTerminator v.3.1 cycle sequencing kit (First BASE, Selangor, Malaysia) and run on an ABI 3730XL Genetic Analyzer (Applied Biosystems Inc., Foster City, CA, USA).

After DNA sequencing, sequence assembly and alignment were conducted using Geneious Prime 2021.1.1 (Kearse et al. 2012). Genetic distance was estimated using the Kimura 2-parameter (K2P) model, implemented in MEGA 7 (Kumar et al. 2016). Phylogenetic analysis based on the COI gene sequences was performed using neighbor-joining (NJ) and Bayesian inference (BI) methods. The NJ tree was built in MEGA 7 with 1000 bootstrap replications (Kumar et al. 2016). The BI tree was constructed in MrBayes v.3.2.7 (Ronquist et al. 2012) and was run for two million generations with sampling every 100 generations and a burnin of 25%. GTR+I was selected as the best-fit model for BI method based on the Akaike Information Criterion (AIC) by using jModelTest v.2.1.10 (Guindon and Gascuel 2003; Darriba et al. 2012). The DNA sequence of *S.asakoae* belonging to the *S.asakaoae* species-group of the subgenus Gomphostilbia was used as the outgroup species. The COI gene sequences deposited in GenBank of *S.* sp. (MT262583), *S.chumpornense* (MT262567, MT262569–MT262570), *S.kuvangkadilokae* (MT262571–MT262573) and *S.piroonae* (MT262574–MT262576) were used for comparison. Newly generated COI gene sequences were registered in GenBank (NCBI) database under the accession numbers: MZ543397–MZ543403.

### ﻿Nomenclature

This paper and the nomenclatural acts have been registered in ZooBank (www.zoobank.org), the official register of the International Commission on Zoological Nomenclature. The Life Science Identifier (LSID) numbers are noted under the new species of black flies.

## ﻿Results

### ﻿Morphological analysis

All females seem to be indistinguishable from one another in many features except the mandible, which had three or four distinct outer teeth in six females (CPPH-1, CPPT-1, CPPT-2, CPPT-3, CPRK-2 and CPRK3), but had a few weak outer teeth in one female (CPRK-1).

All females of *S.* sp. have the subcosta lacking hairs ventrally indicating that these females are placed in the *S.chumpornense* subgroup in the *S.varicorne* species-group. Among six known species of the *S.chumpornense* subgroup, *S.kuvangkadilokae* and *S.piroonae*, both from Thailand, are similar to *S.* sp. in having the hind tibia darkened on the apical half. However, *S.* sp. is distinguished from *S.kuvangkadilokae* by the width of the head relative to the greatest width (4.21–4.66 versus 3.78–4.05), length of the labrum relative to the clypeus (0.65–0.69 versus 0.57–0.59), and length of the fore basitarsus relative to its greatest width (6.29–6.38 versus 5.56); from *S.piroonae* by the length of the sensory vesicle relative to the third segment (0.33–0.39 versus 0.25–0.30), and length of the fore basitarsus relative to its greatest width (6.29–6.38 versus 5.54–5.68) (Takaoka and Srisuka 2010; Takaoka et al. 2014).

### ﻿Genetic analysis

**Figure 1.**
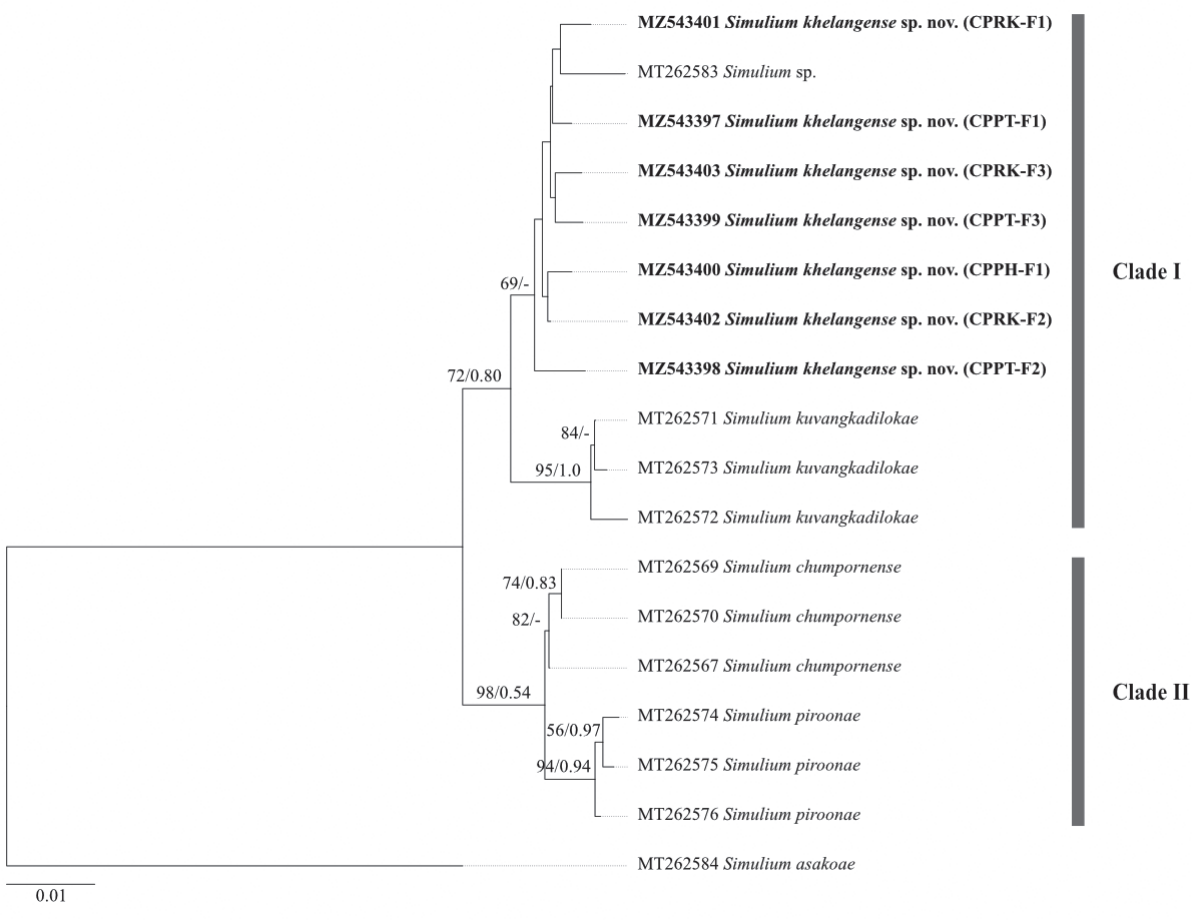
Neighbor-joining tree of the four Thai species in the *S.chumpornense* subgroup of the *S.varicorne* species-group based on 658 bp COI gene sequences. Bootstrap and posterior probability values (NJ/BI) are shown above each branch. The scale bar represents 0.01 substitutions per nucleotide position. Sequences in bold type are generated in this study.

A genetic analysis using the COI gene sequences shows two clear clades, one consisting of *S.kuvangkadilokae* and *S.* sp. including the sample previously reported (MT262583), and the other consisting of *S.chumpornense* and *S.piroonae* (Fig. 1). Genetic analysis similarly shows that *S.* sp. is most closely related to *S.kuvangkadilokae*, with a genetic distance of 1.23–2.81%. Intraspecific divergence for *S.* sp. ranged from 0.30 to 1.54%. Considering the morphological and genetic evidence, we conclude that *S.* sp. is new to science, thus being described here.

### ﻿Descriptions of new species

#### ﻿ Simulium (Gomphostilbia) khelangense

Taxon classificationAnimaliaDipteraSimuliidae

Takaoka, Srisuka & Saeung sp. nov.

83D6204F-6303-5777-ACA0-47D0C09EC0CE

http://zoobank.org/A2B76F93-9D05-4CA7-A03C-2FB789155495

##### Material examined.

***Holotype***: Female (whole body) captured by a sweeping net, at Ban Lek, Fang District, Chiang Mai Province, 20°04'36.3"N, 99°10'53.0"E, 1571 m in elevation, 29 III 2018, by Wichai Srisuka (Site 2). ***Paratypes***: One female and one female (except thorax), same data and date as for the holotype, three females (except thorax), collected at Pratoo Pha, Mueang, Lampang Province (Site 1); three females (except thorax) collected at Ban Romklao Botanic Garden, Chat Trakan, Phitsanulok Province (Site 3).

##### Diagnosis.

Female adult: the only species of the *S.chumpornense* subgroup with antenna with eight flagellomeres, pleural membrane bare, subcosta bare, and hind tibia darkened on apical half, with dark subbasal marking and relatively slender fore basitarsus (6.29–6.38 times as long as its greatest width).

##### Description.

**Female** (*N* = 9). Body length 2.3–2.5 mm.

**Figure 2.**
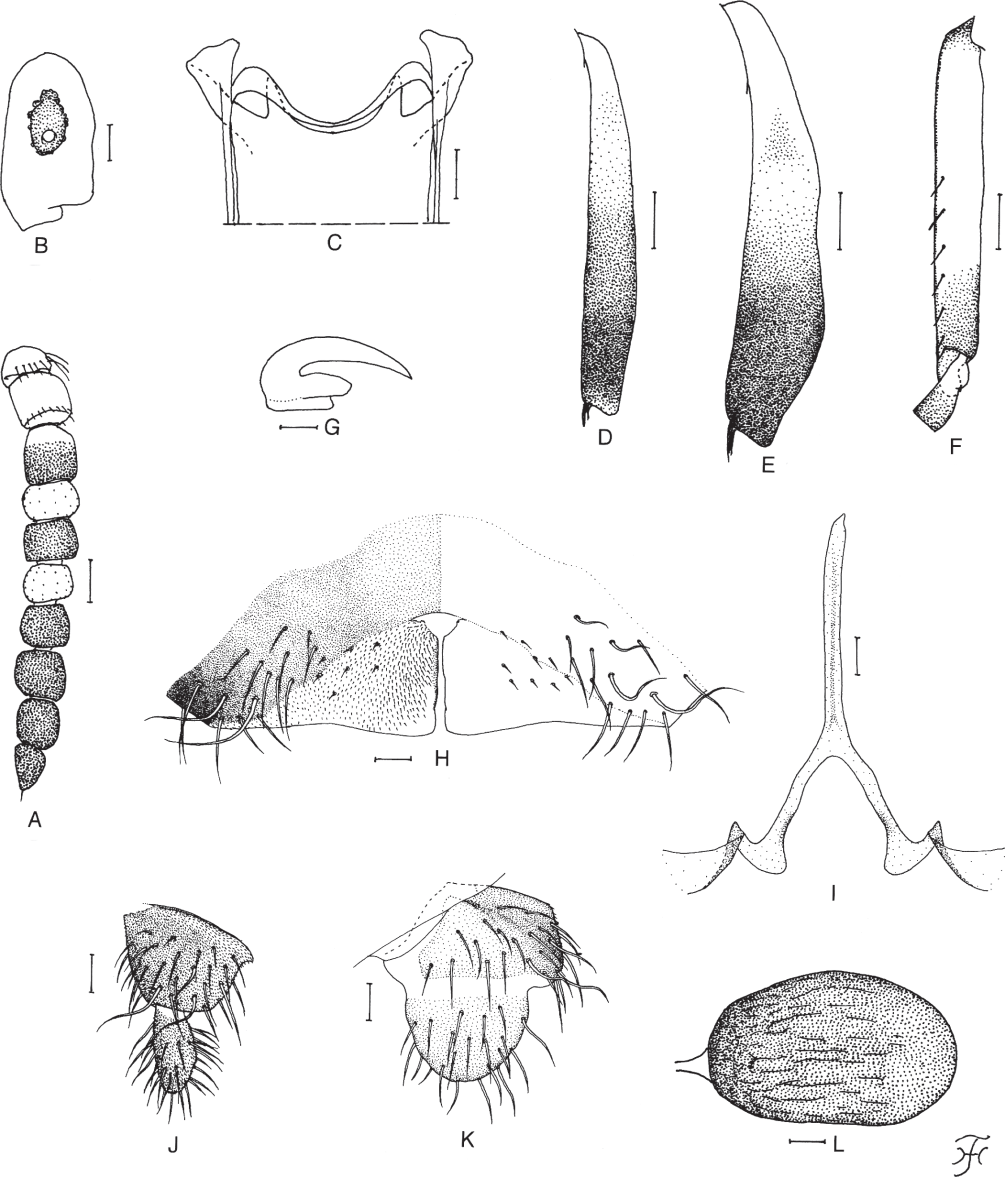
Female of *Simuliumkhelangense* sp. nov. **A** antenna (left side; dorsal view) **B** third palpal segment with sensory vesicle (right side; front view) **C** cibarium **D** mid tibia (left side; outer view) **E** hind tibia (left side; outer view) **F** hind basitarsus and second tarsomere (left side; outer view) **G** claw of hind tarsus (lateral view) **H** eighth sternite and ovipositor valves (ventral view) **I** genital fork (ventral view) **J, K** paraprocts and cerci (right side; **J** ventral view **K** lateral view) **L** spermatheca. Scale bars: 0.1 mm (**D–F**); 0.05 mm (**A**); 0.02 mm (**B, C, H–L**); 0.01 mm (**G**).

***Head*.** Slightly narrower than thorax. Frons brownish black, dull, densely covered with yellowish-white scale-like recumbent short hairs; frontal ratio 1.35–1.44:1.00:1.71–2.09; frons:head ratio 1.00:4.21–4.66. Fronto-ocular area well developed, directed laterally and slightly upward. Clypeus brownish black, densely covered with yellowish-white scale-like short hairs interspersed with several dark unbranched longer hairs along lateral margin on each side. Labrum 0.65–0.69 times as long as clypeus. Antenna (Fig. 2A) composed of scape, pedicel and eight flagellomeres, dark brown to brownish black except scape, pedicel and base of first flagellomere yellowish white, rest of first flagellomere and third flagellomere medium to dark brown, and second and fourth flagellomeres yellow to dark yellow (sometimes light brown). Maxillary palpus composed of five segments, light brown, proportional lengths of third, fourth and fifth segments 1.00:1.00–1.03:2.34–2.48; third segment (Fig. 2B) somewhat swollen apically; sensory vesicle (Fig. 2B) ellipsoidal, 0.33–0.39 times length of third segment, with medium-sized opening. Maxillary lacinia with 9–12 inner, and 12–14 outer, teeth. Mandible with 20–22 inner teeth and with three or four outer teeth at some distance from apex, though outer teeth very weakly developed in one female. Cibarium (Fig. 2C) with pair of short stout submedian projections directed dorsally on dorsal margin.

***Thorax*.** Scutum brownish black (except anterolateral calli ochreous), shiny, gray-pruinose with three longitudinal nonpruinose vittae (one medial and two submedial), densely covered with yellowish-white scale-like recumbent short hairs intermixed with brownish similar hairs. Scutellum dark brown, covered with yellowish-white short hairs and dark brown upright long hairs. Postnotum dark brown, bare, slightly shiny and gray-pruinose when illuminated at certain angle. Pleural membrane bare. Katepisternum dark brown, longer than deep, moderately covered with yellowish fine hairs interspersed with dark brown hairs.

***Legs*.** Foreleg: coxa and trochanter yellowish white; femur medium brown though apical tip yellow; tibia medium brown, except base yellow, and median large portion on outer surface and apex light brown; tarsus brownish black, with moderate dorsal hair crest; basitarsus somewhat dilated, 6.29–6.38 times as long as its greatest width. Midleg: coxa dark brown; trochanter light brown; femur medium to dark brown though apical tip yellow; tibia (Fig. 2D) light brown on basal two-fifths except base yellow and with or without faint subbasal dark marking, and medium to dark brown on apical three-fifths; tarsus light brown except basal three-fourth of basitarsus, basal half of second tarsomere and base of third tarsomere yellowish white. Hind leg: coxa dark brown; trochanter yellowish; femur dark brown with base and apical tip yellowish; tibia (Fig. 2E) dark brown to brownish black on apical half, and yellowish on base, with distinct medium brown subbasal marking (though dark yellow to light brown between subbasal marking and dark apical half, and sometimes subbasal dark marking connected along posterior margin to dark apical half); tibia densely covered with whitish-yellow fine hairs on outer and posterior surface of basal three-fourths; tarsus medium brown except little more than basal two-thirds (though base light brown) of basitarsus and basal half of second tarsomere yellowish white; basitarsus (Fig. 2F) narrow, nearly parallel-sided, 6.55–7.05 times as long as wide, and 0.58–0.61 and 0.48–0.52 times as wide as greatest width of tibia and femur, respectively; calcipala (Fig. 2F) 1.3 times as long as wide, and 0.45–0.47 times as wide as width of basitarsus; pedisulcus (Fig. 2F) well marked. Hind tarsal claw (Fig. 2G) with large basal tooth 0.46–0.47 times length of claw.

***Wing*.** Length 2.0 mm. Costa with dark brown spinules and dark brown hairs except basal portion with patch of white hairs. Subcosta bare. Hair tuft on base of radial vein white. Basal portion of radius fully haired. Basal cell absent.

***Halter*.** White with base of stem darkened.

***Abdomen*.** Basal scale light brown, with fringe of yellowish-white fine hairs. Dorsal surface of abdomen medium brown to brownish black except little less than basal one-half lighter, moderately covered with yellowish-white short hairs interspersed with dark brown long hairs; tergites of segments 2 and 6–8 shiny; sternal plate on segment 7 undeveloped.

***Genitalia*.** Sternite 8 (Fig. 2H) bare medially, with 14–16 long stout hairs and two to five short setae on each side. Ovipositor valves (Fig. 2H) nearly triangular, thin, membranous, each moderately covered with microsetae interspersed with five or six short setae; inner margins slightly sinuous, moderately sclerotized. Genital fork (Fig. 2I) of usual inverted-Y form, with narrow arms; arm folded medially. Paraproct in ventral view (Fig. 2J) rounded outwardly and tapered medially, with 26–31 long hairs on ventral and lateral surfaces, and with five sensilla on anteromedial surface; paraproct in lateral view (Fig. 2K) moderately produced ventrally beyond ventral margin of cercus, 0.58–0.68 times as long as wide. Cercus in lateral view (Fig. 2K) rounded posteriorly, 0.44–0.68 times as long as wide. Spermatheca (Fig. 2L) ellipsoidal, 1.67–1.88 times as long as wide, well sclerotized except duct unsclerotized, and with many fissures on surface; internal setae absent; both accessory tubes slender, slightly larger in diameter than major one.

**Male, pupa and larva.** Unknown.

##### Etymology.

The species name *khelangense* refers to Khelang, an old name of Lampang Province, where this new species was collected.

##### Distribution.

Thailand (Lampang, Phitsanulok and Chiang Mai).

##### Ecological note.

Females of this new species were captured while attracted to a human, though they have a large claw tooth, a characteristic suggesting that this species is ornithophilic (Adler et al. 2004).

##### Discussion.

*Simuliumkhelangense* sp. nov. is placed in the *varicorne* species-group in the subgenus Gomphostilbia by having the antenna with eight flagellomeres (Fig. 2A). It is further placed in the *chumpornense* subgroup by having the pleural membrane bare, and female subcosta lacking hairs ventrally, as defined by Takaoka (2012).

The female of this new species is distinguished from those of *S.kuvangkadilokae* and *S.piroonae* of the *S.chumpornense* subgroup, as noted above. This species is also distinguished from the four other members of the same subgroup: *S.chumpornense* from Thailand, *S.sumbaense* Takaoka & Suana from Sumba, Indonesia, *S.tomae* Takaoka from Sulawesi, Indonesia, and *S.varicorne* Edwards from Sumatra and Java, Indonesia, and Peninsular Malaysia, by the hind tibia darkened on the apical half (darkened on the apical one-third in the latter four species) (Kuvangkadilok and Takaoka 2000; Takaoka 2003; Takaoka et al. 2018a, b).

### ﻿Key to females of 14 species of the varicorne species-group of the subgenus Gomphostilbia

The female of *S.breviflagellum* Takaoka & Sofian-Azirun from Vietnam is not included because its female is unknown.

**Table:** 

1	Antenna with seven flagellomeres	**2**
–	Antenna with eight flagellomeres	**3**
2	Sensory vesicle 0.29–0.31 times length of third palpal segment	***S.charlesi* Takaoka**
–	Sensory vesicle 0.21–0.25 times length of third palpal segment	***S.novemarticulatum* Takaoka & Davies**
3	Pleural membrane haired	***S.trirugosum* Davies & Györkös**
–	Pleural membrane bare	**4**
4	Subcosta haired ventrally	**5**
–	Subcosta bare	**8**
5	Abdominal segments 5–8 shiny dorsally	**6**
–	Abdominal segments 6–8 shiny dorsally	**7**
6	Flagellomeres darkened except basal one-third of first flagellomere yellow	***S.huangi* Takaoka**
–	Flagellomeres 3 and 5–8 darkened and others yellow	***S.burtoni* Takaoka & Davies**
7	Hind femur entirely darkened	***S.shogakii* Rubtsov**
–	Hind femur darkened on apical one-third	***S.synanceium* Chen & Cao**
8	Hind tibia darkened on apical half	**9**
–	Hind tibia darkened on apical one-third	**11**
9	Fore basitarsus 6.29–6.38 times as long as its greatest width	***S.khelangense* sp. nov.**
–	Fore basitarsus 5.54–5.68 times as long as its greatest width	**10**
10	Head 3.78–4.05 times as wide as greatest width of frons	***S.kuvangkadilokae* Pramual & Tangkawanit**
–	Head 4.30–4.54 times as wide as greatest width of frons	***S.piroonae* Takaoka & Srisuka**
11	Head 6.7 times as wide as greatest width of frons	***S.tomae* Takaoka**
–	Head 3.7–5.2 as wide as greatest width of frons	**12**
12	Head 4.7–5.2 times as wide as greatest width of frons	***S.varicorne* Edwards**
–	Head 3.9–4.0 as wide as greatest width of frons	**13**
13	Height of frons 1.7 times as long as narrowest width	***S.chumpornense* Takaoka & Kuvangkadilok**
–	Height of frons 1.3–1.4 times as long as narrowest width	***S.sumbaense* Takaoka & Suana**

## ﻿Conclusions

Considering the morphological and genetic evidence, we conclude that S.sp.sensuAupalee et al. (2020), is new to science, thus being described here. Females of S. (G.) khelangense sp. nov. were captured while attracted to a human. This new species is distributed in northern and central Thailand.

## Supplementary Material

XML Treatment for Simulium (Gomphostilbia) khelangense
